# Vascular suture line wrapping for Aortoiliac anastomoses following open surgical repair of Infrarenal Behçet’s Aortoiliac aneurysms

**DOI:** 10.1186/s13023-019-1048-y

**Published:** 2019-04-15

**Authors:** Ahmed Mousa, Ibrahim Hanbal, Alaa Sharabi, Mohammed A. Nasr, Abdelfattah K. Nassar, Mai A. Elkalla

**Affiliations:** 1 Department of Vascular & Endovascular Surgery, Al-Hussain University Hospital, Faculty of Medicine for Males, Al-Azhar University, Darrasa, Cairo, 11675 Egypt; 2 Division of Vascular & Endovascular Surgery, Department of Surgery, College of Medicine, King Faisal University, Eastern Province, Al-Ahsa 31982 Saudi Arabia; 3 Division of Vascular & Endovascular Surgery, Department of Surgery, Faculty of Medicine, Al-Azhar University, Assiut Branch, Assiut, Egypt; 4 Department of Rheumatology and Rehabilitation, Al-Hussain University Hospital, Faculty of Medicine for Males, Al-Azhar University, Cairo, Egypt; 5 Medical Student, Faculty of Medicine, Helwan University, Cairo, Egypt

**Keywords:** Behçet’s aortoiliac aneurysms, Heparin-bonded Dacron® graft, Anastomotic pseudoaneurysms, Vascular anastomosis, Prophylactic prosthetic mechanical wrapping

## Abstract

**Background:**

This study was conducted to evaluate our local experiences of adjunctive mechanical prosthetic wrapping for aortoiliac vascular anastomoses as a prophylactic measure following surgical repair of Behçet’s aortoiliac aneurysms. The goal of prosthetic wrapping to reinforce the vascular anastomoses by mechanical protection to reduce the bleeding complications, and consequently pseudoaneurysm formation. This was aided by the administration of pre- and postoperative immunosuppressive therapy as an adjuvant treatment.

**Methods:**

A seven-year retrospective study was conducted between January 2006 and December 2012, retrieving data of patients with Behçet’s aortoiliac aneurysms. All patients underwent open surgical repair using a heparin-bonded synthetic Dacron® graft. Data for all patients were retrieved and analyzed for diagnostic procedures, graft selection, as well as, different methods of surgical repair. Graft-related complications such as anastomotic pseudoaneurysms, occlusion, and thrombosis were also reported.

**Results:**

Sixteen patients were recruited in this study. There were  11 (69%) males and 5 (31%) females with the male to female ratio 2:1. The patients’ age ranged between 25 and 47 years with the mean of 36.4 ± 7.3. All Behçet’s aortic/aortoiliac aneurysms were repaired by the application of heparin-bonded Dacron® tube and bifurcated grafts. The anastomotic wrapping technique was performed for both the proximal and the distal vascular anastomoses. The technical success of aortoiliac aneurysm and wrapping techniques was achieved in 100% of patients. All patients were given pre- and postoperative systemic immunosuppressive therapy. No graft-related complications were reported except for only one anastomotic pseudoaneurysm that developed at one of the right iliac anastomoses, that developed within 24 months after follow up.

**Conclusions:**

Mechanical prosthetic wrapping for vascular anastomoses in patients with Behçet’s aortic/aortoiliac aneurysms is a feasible, simple, and reliable technique with low morbidity and mortality. It was performed as a prophylactic measure to avoid the development of postoperative anastomotic pseudoaneurysms. It must be performed for all patients with Behçet’s arterial aneurysms whenever possible. Furthermore, the supplemental administration of pre- and postoperative systemic immunosuppressive therapy should be considered as an important factor for the prophylaxis and prevention of anastomotic pseudoaneurysms and other graft-related complications.

## Background

Behçet’s disease is a chronic, multisystemic, inflammatory rare syndrome with exacerbations and remissions of unexpected duration. The health problems related to Behçet’s disease result from systemic inflammation of blood vessels (vasculitis). This inflammation generally affects small blood vessels in the eyes, mouth, genitals, and skin, in addition to blood vessel involvement [1]. It is manifested by skin lesions (e.g., pseudofolliculitis, erythema nodosum, acneiform nodules, papulopustular lesions, and superficial thrombophlebitis); recurrent genital and oral aphthous ulceration, as well as eye lesions (e.g., retinal vasculitis, anterior and posterior uveitis). In addition to gastrointestinal tract involvement (e.g., ulcers of the cecum and terminal ileum); epididymitis, arthritis, neurological, pulmonary, and cardiovascular lesions are also reported. Occlusive lesions and aneurysms are formed in the arteries and thrombophlebitis in the veins [2, 3]. Vascular involvement affects approximately 1.8–51.6% of Behçet’s disease patients. All arteries and veins of different sizes are affected. Venous manifestations include thrombophlebitis and lower extremity deep venous thrombosis (DVT), which may extend to affect the superior and inferior vena cava [4, 5]. It has been shown that, dural sinus involvement is associated with large vessel disease, while DVT is associated with pulmonary artery aneurysms among 90% of patients. The incidence of arterial lesions in Behçet’s syndrome varies between 2.2–18% of affected patients [6–8]. Most literature reported that arterial lesions are isolated, while multiple lesions are not frequently encountered [9]. The most often reported arterial complications are occlusion and aneurysm formation. Whereas the most commonly involved arteries are the aorta either abdominal or thoracic. Although however, the pulmonary, femoral, subclavian, popliteal, carotid as well as, the coronary arteries may also be affected. Bypass surgery was considered the treatment of a choice for the repair of Behçet’s disease-associated aortoiliac and other peripheral artery aneurysms. Furthermore, extra-anatomical bypass or the use of a normal arterial segment as an interposition graft has been also reported [10]. Moreover, the use of an endovascular covered stent may be a suitable method of aortoiliac aneurysm repair [11]. In addition to operative aneurysm repair and to prevent the development of postoperative pseudoaneurysms and other graft-related complications, pre- and postoperative systemic immunosuppressive agents is recommended, as an adjunctive treatment [12–14]. While anastomosing graft to host artery, vascular suture lines has been reinforced with expanded polyethylene terephthalate (ePTE - Dacron®), polytetrafluoroethylene (ePTFE), omentum, an autogenous vein, or mesh to wrap the vascular anastomoses. This technique was performed to reduce the rate of postoperative hemorrhage and avoid  slipping of the vascular ligatures, thereby preventing the associated complications, especially, anastomotic pseudoaneurysms [14, 15]. In this article, we reported our local experiences on the prosthetic wrapping technique for the aortoiliac anastomoses in Behçet’s aortic/aortoiliac aneurysms using a heparin-bonded Dacron® as a patch graft to prevent anastomotic site pseudoaneurysms. This wrapping technique was performed as a prophylactic measure. However, the administration of pre- and postoperative immunosuppressive therapy as an adjuvant treatment is mandatory to prevent postoperative anastomotic pseudoaneurysms and other Behçet’s disease-related complications.

## Methods

**Fig. 1:**
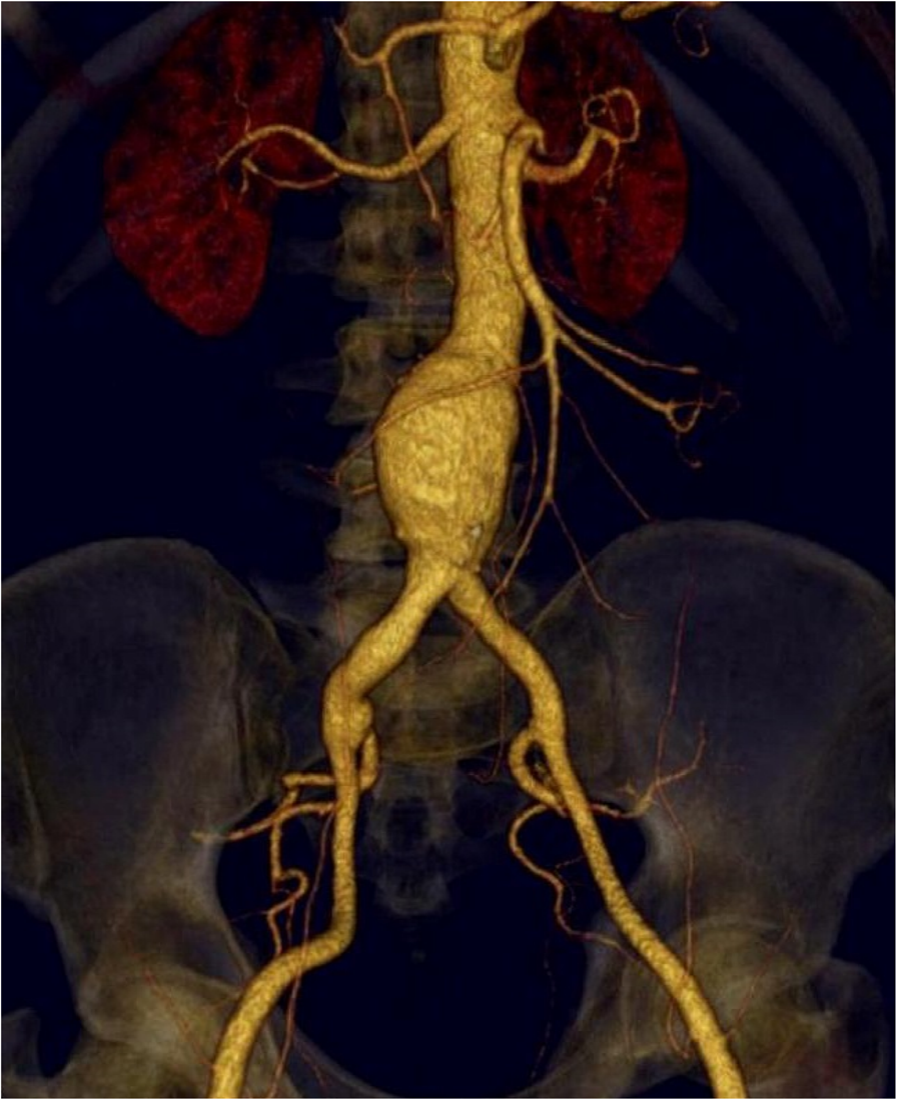
CTA showing infrarenal abdominal aortic and iliac aneurysms

A seven-year retrospective descriptive study took place; from January 2006 to December 2012, and after approval of our Institutes’ Review Board (IRB) and ethical committee, the data of Behçet’s disease-associated aortoiliac aneurysms were reported. A thorough study of all patients´ files retrieving the demographics, clinical data, perioperative preparation, utilized anesthesia, the different kinds of surgical repair, as well as long-term postoperative complications. Different imaging modalities including color Doppler ultrasonography (CDUS), computed tomography angiography (CTA) scanning (Fig. 1) were also reported. All Behçet’s disease diagnosed patients were referred to the vascular surgery department in our institutes from either rheumatology or dermatology departments. The diagnosis of Behçet’s disease was performed according to the international standard criteria for Behçet’s disease [16]. This study included all patients with infrarenal aortic/aortoiliac aneurysms while, patients with thoraco-abdominal aortic aneurysms, carotid and peripheral arterial aneurysms, as well as, patients with venous system involvement were excluded from the study.

### Preoperative medical preparation

Systemic medications were given in the form of azathioprine 50–100 mg/day, as an immunosuppressive agent/colchicine in a dosage of 1.2 mg/day. The dose of immunosuppressive therapy was adjusted according to the level of erythrocyte sedimentation rate with the reference range < 20 mm/h [17, 18]. Corticosteroid therapy was supplemented 5–60 mg/day and gradually tapered by 10–20 mg/month.

### Graft selection and operative aneurysm repair

**Fig. 2:**
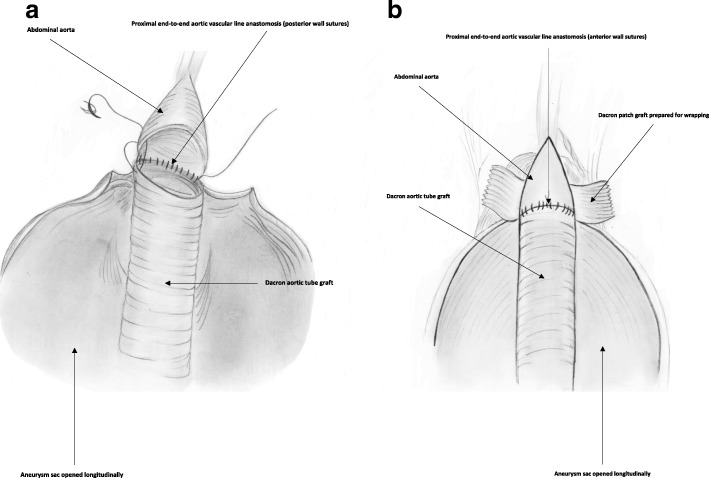
Proximal posterior (**a**) and anterior (**b**) wall aortic anastomosis

**Fig. 3:**
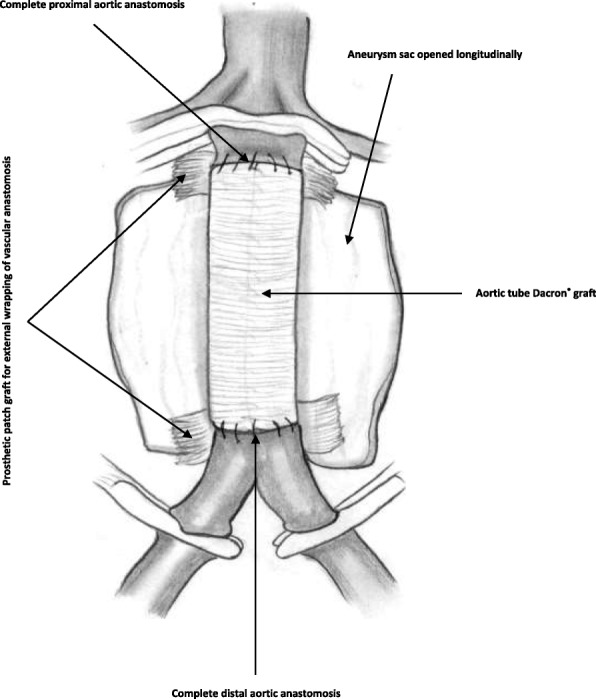
Anastomosis of the proximal and the distal aortic tube Dacron® graft

**Fig. 4:**
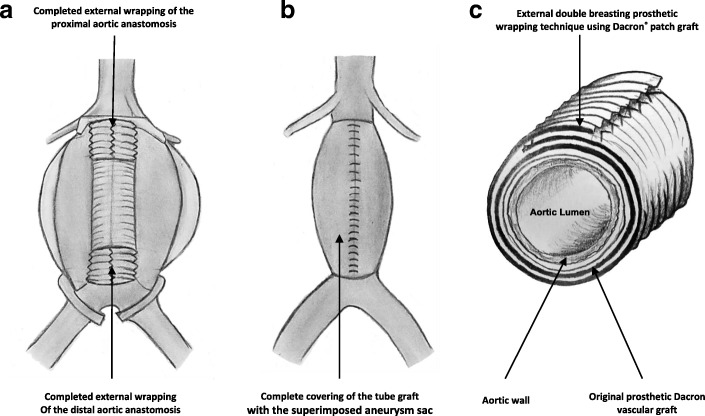
(**a**) Complete wrapping of both the proximal and distal aortic anastomosis; (**b**) the overlying aneurysm sac and the retroperitoneum are closed to cover the prosthetic bypass; (**c**) 3-D cross-section demonstrating the wrapping technique

**Fig. 5:**
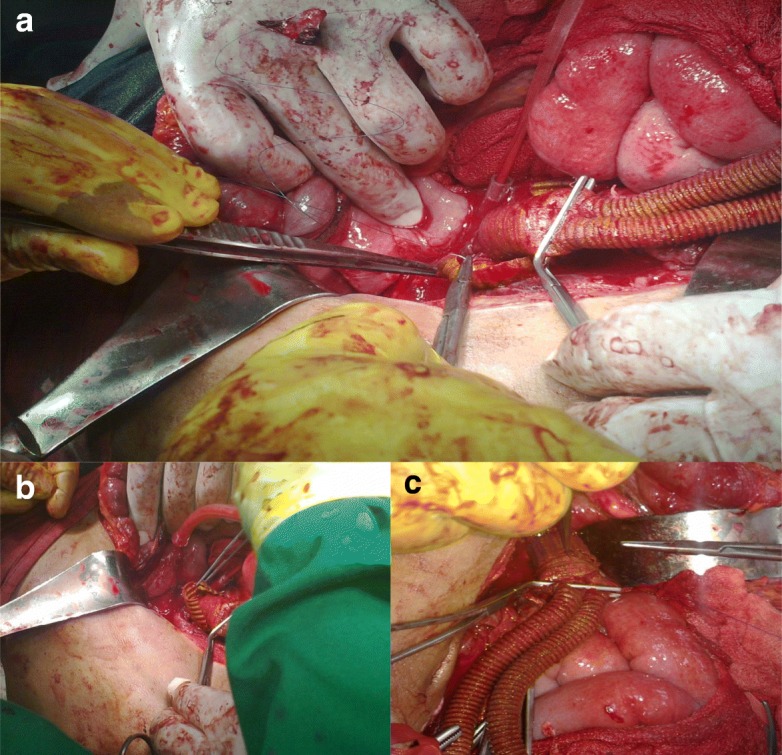
Technique of proximal aortic anastomotic full prosthetic wrapping; (**a**) prosthetic Dacron graft encircling the proximal aortic anastomosis; (**b**) suturing of the wrapped graft in a double breasting technique; (**c**) final picture: complete vascular suture line graft wrapping

**Fig. 6:**
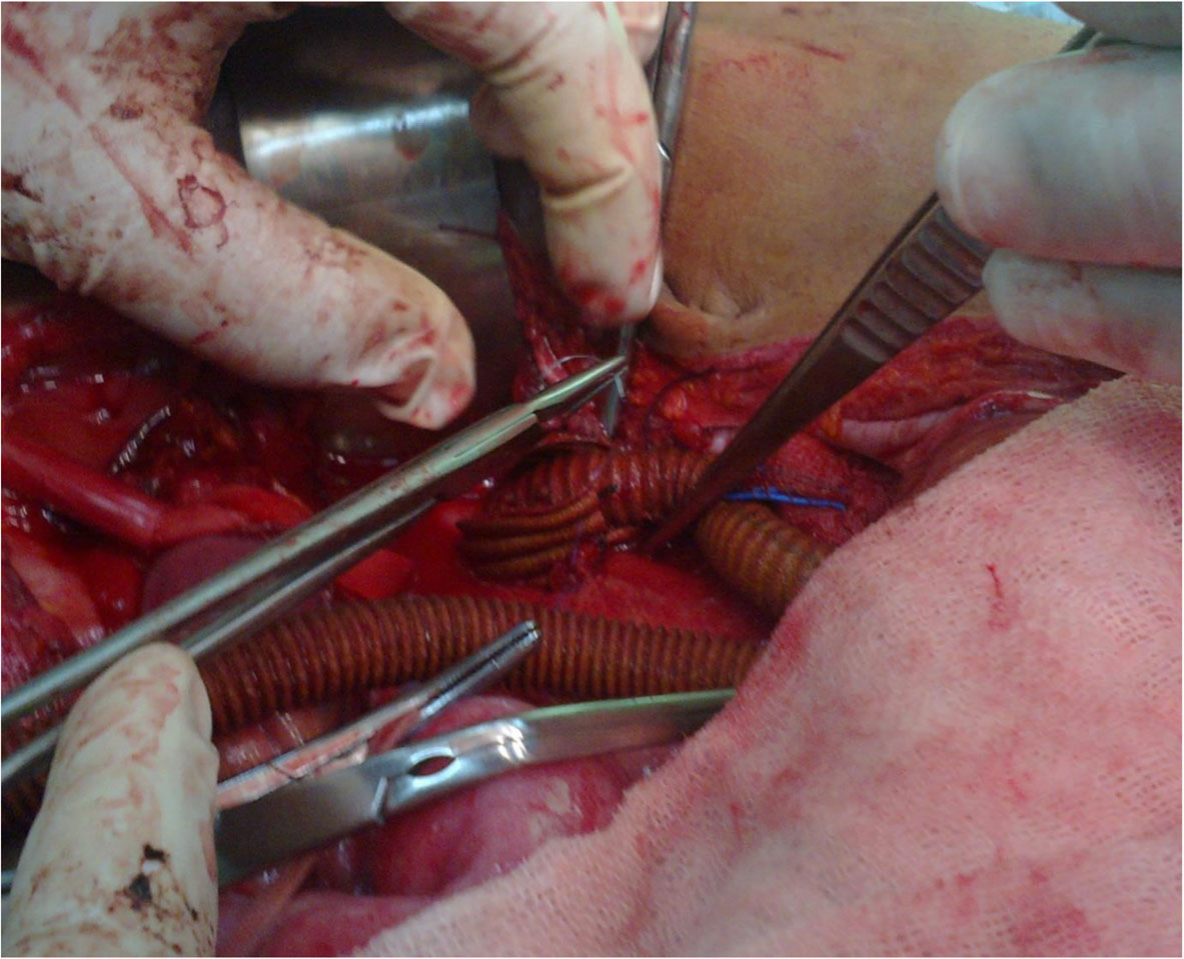
Distal end-to-end aortoiliac anastomotic external wrapping

**Fig. 7:**
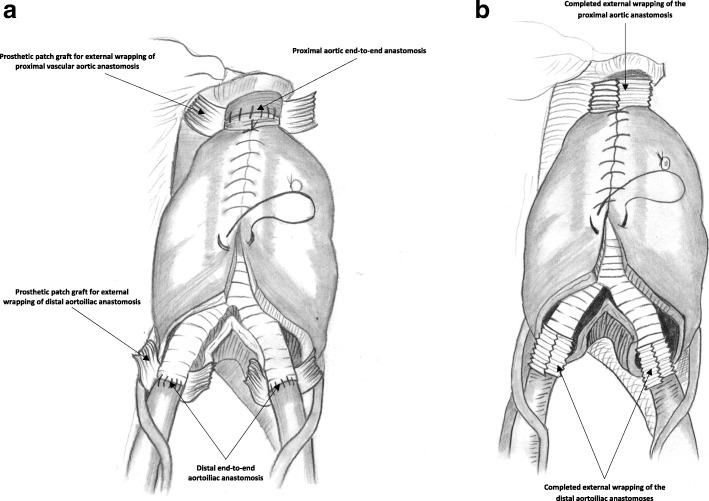
Aorto-bi-iliac bypass graft using end-to-end proximal and distal anastomoses; (**a**) preparation of the prosthetic Dacron® patch graft around the poroximal aortic and the distal aortoiliac anastomoses; (**b**) complete wrapping of the proximal aortic and the distal aortoiliac anastomoses

Surgical repair of aortic/aortoiliac aneurysms was performed using a heparin-bonded Dacron® grafts, which is ePET, heparin-bonded Dacron® [Inter Gard heparin-impregnated Dacron® grafts (MAQUET Holding GmbH & Co. KG., Rastatt, Germany)]. The choice of the implanted graft for repair of aortic/aortoiliac aneurysms was based on, the improving outcomes when using synthetic prosthesis bonding anticoagulants. Using Carmeda BioActive Surface (CBAS) technology, the heparin-coated Dacron® prosthesis has heparin-bonded onto the luminal surface of the ePET graft, which immobilizes the heparin molecule with a single covalent bond that does not alter its anticoagulant properties [19]. Five patients with an isolated AAA had initially received an interposition tube graft replacement. On the other hand, 11  patients with concomitant aortoiliac aneurysms were treated by the administration of bifurcated Y-shaped graft. The size of the tube grafts ranged between 18 and 20 mm. Moreover, the size of either 18 by 9 mm or 16 by 8 mm is typically chosen for male patients. While a size of either 14 by 7 mm or even 12 by 6 mm are usually suitable for female patients. Systemic administration of unfractionated heparin was routinely given to all patients in a dosage of 100 IU/kg, followed by clamping of the proximal and distal parts of the aneurysm. The aneurysm sac was opened longitudinally and a tube graft was utilized to reconstruct the aorta in cases of an isolated AAA**.** The proximal aorta was completely transected to provide free circumferential cuff**s** for graft anastomosis and to allow for proper application of the prosthetic wrapping material to fully surround the anastomosis. Anastomoses of proximal (Figs. 2a-2b) and distal aortic tube graft to host artery were performed in an end-to-end fashion using a running 5–0 polypropylene suture (Ethicon, Somerville, NJ) (Fig. 3)**.** Moreover, both anastomoses were reinforced with a synthetic patch graft wrapped externally around the vascular anastomoses (Figs. 4a-4c and 5a-5c). In cases of concomitant aortoiliac aneurysms, a Y-shaped bifurcated graft was utilized. Its proximal end was anastomosed to the proximal aorta using end-to-end anastomosis. While its distal ends were anastomosed to the external iliac arteries on both sides (i.e. aorto-bi-iliac bypass) using the end-to-end configuration for all patients. The wrapping technique was performed using a vertical mattress sutures (double breasting technique) using 4–0 polypropylene for the proximal and 5-0 polypropylene for the distal vascular suture lines for each limb of the bifurcated graft (Figs. 6 and 7a-7b).

### Postoperative follow up

Follow up was performed within two weeks after patients’ discharge, then every month for the next six months, and then every 12 months for the next 60 months. The mean follow-up period was 28.93 ± 21.68 months, ranging from 1 to 60 months. All patients were called back to the outpatient vascular clinic for systematic evaluation. Formal evaluation included routine physical examination, local vascular examination of the limbs for clinical improvement, as well as disease regression/progression. Diagnostic CDUS/CTA was carried out each visit for hemodynamic improvement to evaluate the graft patency, recurrent aneurysms, and other graft-related complications.

## Results

Within a seven-year duration from January 2006 to December 2012, sixteen patients with Behçet’s aortic/aortoiliac aneurysms were surgically treated. They included 11 (69%) males and 5 (31%) females with a male to female ratio of 2:1. The patients’mean age was 34 ± 7.7, ranging from 23 to 45 years. Patients’ demographic data and clinical presentations are listed in (Table 1). All the studied patients were presented with infrarenal aortic/aortoiliac aneurysms. Most of them, 69% (*n* = 11) showed concomitant infrarenal AAA and iliac artery aneurysms limited to the common iliac arteries. On the other hand, the remaining 31% (*n* = 5) were presented with an isolated infrarenal AAA. All aneurysms were saccular in shape except for only two patients whose aneurysms were diagnosed as fusiform in shape. Their sizes varied from 4.9–10.2 cm, with a mean diameter of (6.5 ± 1.59 cm). All patients underwent elective open surgical repair, except for only one patient who presented with a life-threatening ruptured aneurysm that necessitated an emergency intervention. Blood transfusion was given preoperatively for only four patients who underwent elective surgery, in addition to the patient who presented with the ruptured aneurysm. The operative time ranged from 105 to 240 min, with a mean of 135 ± 44. Ten patients underwent strict observation in the intensive care unit (ICU) for a period of 2–3 days. Operative technical success was obtained in 100% of patients without any complications. The types of operative procedures are illustrated in (Table 1).

**Table 1:** Patients’ demographics, clinical presentation, and different types of AAA repair

Factor	Results		
Age			
Age in years (mean)	34 ± 7.7		
Min - Max	23–45		
Gender	n	%	Total (n & %)
Male	11	69.00%	(16) 100%
Female	5	31.00%	
Clinical presentation			
Asymptomatic aneurysms (size ≥5.5 cm)	5	31.25	(16) 100%
Pulsatile firm abdominal mass ≥ 5.5 cm	3	18.75	
Painful abdominal swelling with back pain	3	18.75	
Distal tiny embolization	1	06.25	
Ruptured AAA	1	06.25	
Claudication	3	18.75	
Types of AAA repair			
Aorto-bi-iliac bypass surgery using Y-shaped bifurcated graft for aortoiliac aneurysms	11	69.00%	(16) 100%
Aortic tube interposition graft for isolated AAA	5	31.00%	

*Min* Minimum; *Max*: Maximum; *AAA* Abdominal Aortic Aneurysm

**Fig. 8:**
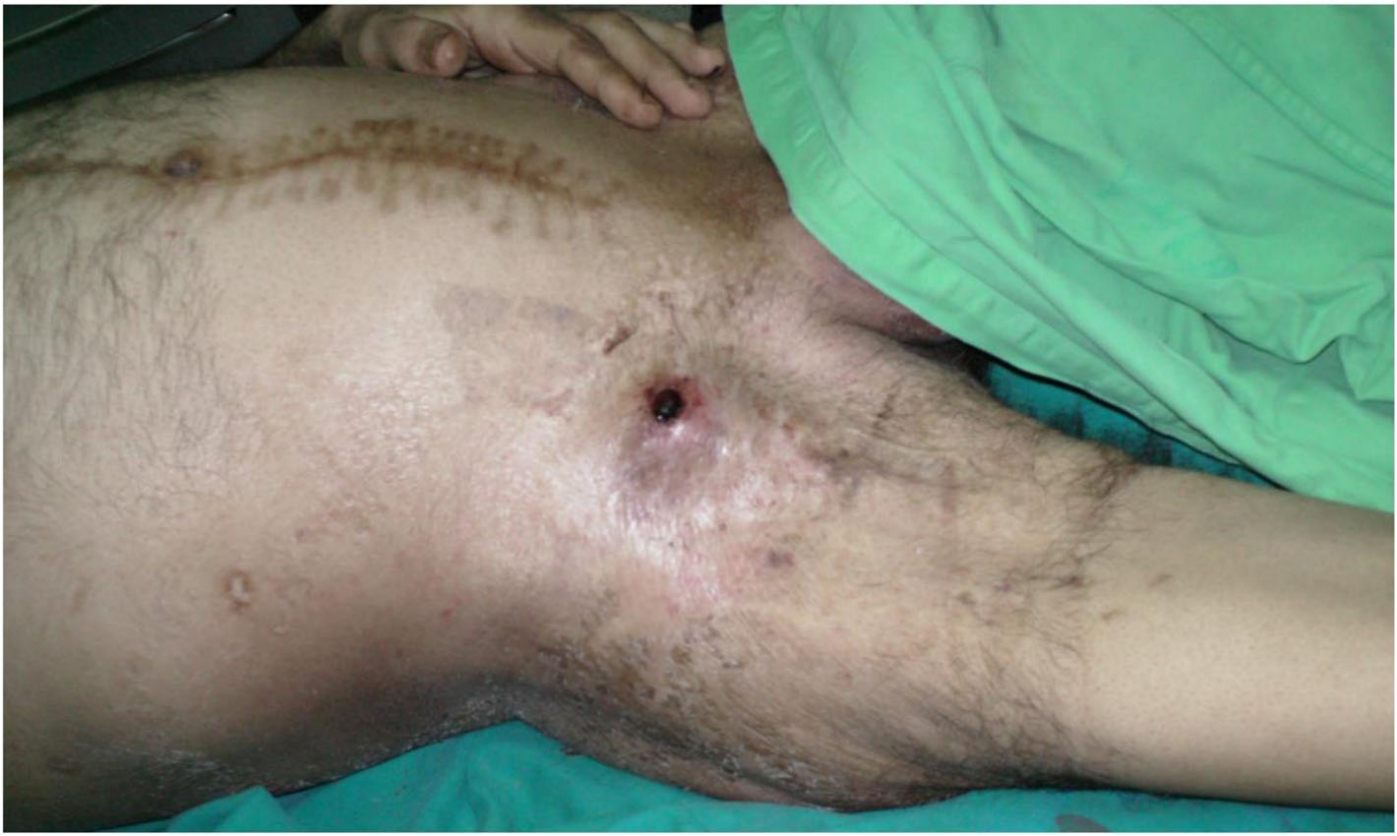
Right groin anastomotic pseudoaneurysm

Furthermore, one anastomotic pseudoaneurysm was developed 24 months after follow-up, in a 37-year old male patient. It was diagnosed clinically as an expansile mass within the right groin (Fig. 8). Its diagnosis was confirmed by CTA. As it was smaller in size, it has been responded well to conservative medical treatment in the form of azathioprine 2 mg/kg/day, in addition to 1.5 mg/kg/day methyl-prednisolone for two weeks duration.

Other graft-related complications such as anastomotic rupture, graft infection and thrombosis were not reported.

### Postoperative medical treatment

All patients were given postoperative medications 4–8 days after surgery to allow for healing of the abdominal wound and to avoid early postoperative wound infection. Corticosteroid injections were given (every other day) as 3 pulses of 1 g prednisolone which continued for one month at a dose of 1 mg/kg/day, then gradually tapered by 10–20 mg/month but, a dose of 5-10 mg/day commonly continued for 1–2 years or more. A total of 12 patients were given cyclophosphamide and the remaining 4 patients treated with azathioprine as well as corticosteroids. Cyclophosphamide was received for a median of 12 months duration usually, in the form of intravenous monthly boluses 1 g, and then shifted to azathioprine for maintenance treatment.

## Discussion

Behçet’s disease may be associated with vascular complications in the form of peripheral arterial aneurysms mostly affecting all arteries [20]. Moreover, aneurysm formation is challenging for most vascular surgeons because of its complicated nature and pathology [21–23]. The use of the endovascular technique is still limited in patients suffering from Behçet’s vascular disease [24]. The literature reported a simple arterial ligation to avoid reconstructive complications. Due to the high probability for the presence of vasculitis in venous tissue, it is not recommended to use autologous venous grafts [25]. The use of synthetic grafts is recommended in a wider range to treat Behçet’s disease-related aortic/aortoiliac aneurysms [26]. This may have a role in decreasing the long-term risk of complications if accompanied by systemic immunosuppressive therapy [27, 28]. This is supported by the recommendations of the European League Against Rheumatism (EULAR) [12, 17]. Aneurysms usually affect all known arteries including, the aorta, iliac, femoral, popliteal, as well as the tibial arteries. Yet, the abdominal aorta is the most commonly involved artery, followed by the pulmonary and femoral arteries [28–30]. Ruptured aneurysms are the most frequently encountered complications and are considered as the most common cause of Behçet’s disease-related mortality [31]. This retrospective analysis reported our local experience of 16 patients presented with Behçet’s aortic/aortoiliac aneurysms. Our patients’ number coincides with that reported in the literature [10, 30, 31]. Our retrieved data reported a marked male predominance with a male to female ratio (2:1), which are similar to that previously reported in the literature [6–8, 20–23, 32]. Infrarenal aortic aneurysms were reported in all of our patients and symptomatic aneurysms were reported in 11 out of 16 patients. This data coincides with previously published reports [10, 22, 25]. In our series, Behçet’s disease-related arterial lesions were encountered in younger age groups (i.e. mean age 34 ± 7.7 years). This data coincides with that reported in the literature [24, 25].

We created arterial anastomosis by using a heparin-bonded Dacron® graft whether tube or bifurcated. We favored the use of a synthetic Dacron® grafts to ePTFE graft, as it is a highly resilient fabric and can be easily manipulated with an excellent healing ability [27]. In addition, the use of Dacron® versus ePTFE graft was reported to have a greater patency rate [33]. We favored the use of an end-to-end vascular anastomosis for the proximal and the distal anastomosis in all patients. Because it provides a better in-line stream pattern with minimal turbulence and more convenient hemodynamic characteristics; as well as lower rates of suture-line false aneurysms and favorable long-term patency rates. This technique coincides to the previous literature reports [25, 34, 35]. Literature evaluation for cost-effectiveness of heparin-coated versus ePTFE grafts reported that heparin-coated Dacron® grafts seems to be highly cost-effective overall ePTFE grafts for revascularization procedures [36, 37]. Prosthetic wrapping technique has been performed for both the proximal and the distal vascular anastomotic lines in all patients to prevent postoperative pseudoaneurysms, which is a common complication of such disease. This technique was similar to that previously reported in the literature [38–42]. We successfully applied a piece of Dacron® strip wrapped for all the vascular anastomoses. The long-term follow up and the reliability of this technique coincides with the previous literature reports [25, 43, 44]. In order to prevent postoperative pseudoaneurysms, some authors advocate the reinforcement of vascular anastomotic suture line with a patch of ePTFE graft [10, 14], synthetic mesh wrapping [45], native aneurysm wall [46], remnant of the natural aortic wall [47], or omental wrapping [15, 24, 25, 39, 48, 49], applied to the outer surface of the vascular anastomoses contradicting our used material. The goal of the use of a prosthetic wrapping is to reinforce and mechanically support the vascular anastomotic suture line thus, reducing the bleeding complication, and consequently pseudoaneurysm formation [42–49].

Our results in comparison to the literature reports are illustrated in (Table 2). We reported a postoperative follow-up period of a maximum of 60 months, contradicting that reported in the literature where they reported a follow-up period reaching up to 84 months [10]. Moreover, in our series, we encountered one anastomotic pseudoaneurysm within 24 months of follow-up. This result contradicting that reported in the literature [50, 51], where anastomotic pseudoaneurysms developed within 6–17 months of follow-up. The development of anastomotic pseudoaneurysm in our series may be related to an associated mechanical, or turbulence/shear, stresses on an anastomosis. By this, prosthetic dilatation may transmit tension to the suture line and adjacent host artery, resulting in anastomotic disruption. Extraneous tension on the anastomosis may result in elastic recoil of the graft, leading to separation from the vessel wall [52, 53]. The developed pseudoaneurysm is generally inflammatory in nature, so it responds well to conservative medical treatment. Our conservative treatment results are similar to that previously reported in the literature [54]. Moreover, postoperative prophylactic antiplatelet and anticoagulant therapy was given to all patients, yet, we did not report any cases of neither graft thrombosis nor occlusion, contradicting that reported in the literature [10, 25, 27, 28, 49–51]. Reasonable use of adjuvant postoperative systemic immunosuppressive and corticosteroid therapies have been suggested as effective preventive measures, to avoid a relapse of Behçet’s disease vascular-related complications [25, 53–56]. The prevalence of Behçet’s disease is nicely demonstrated in the literature. They reported that it is a worldwide disease but, mainly affects people living around the Mediterranean basin and in Japan. Turkey has the highest prevalence at 370 to 420 cases per 100,000 populations [57, 58], whereas Western countries have a much lower prevalence of 5 per 100.000 [59]. Behçet’s disease typically presents in young adults aged 20 to 40 years, and intimate relation with HLA-B51 has been demonstrated. Infection with hepatitis C virus, Parvovirus B19, Herpes simplex virus, and *Streptococcal Sanguis* has been involved and predispose to the development of Behçet’s disease [60]. The most significant disadvantage of our study is the small number of cases; however, as the disease is very uncommon our clinical experience is considerable.

**Table 2:** Our results in comparison to the literature reports or vice versa

	Current Study	Kalko et al. [10]	Kwo et al. [25]	Le Thi Huong et al. [27]	Tuzun et al. [28]	Hosaka et al. [48]	Unal et al. [52]	Nitecki et al. [54]	Ascione et al. [60]	Shen et al. [61]	Koksoy et al. [62]	Iscan et al. [63]	Ozeren et al. [64]	Saadoun et al. [65]	Skourtis et al. [66]
Number of patients	16	16	12	25	15	10	11	18	1	8	23	20	12	101	45
Peripheral aneurysms	16	12	12	25	18	10	11	8	1 (100%)	8	29	0	9	101	49
Anastomotic pseudoaneurysms	1 (6.25%)	2 (17%)	0	N/A	4 (22%)	5 (50%)	6 (54%)	1 (12.5%)	2 (200%)	1 (12.5%)	7 (24%)	20 (100%)	1 (11%)	0	14 (28.5%)
Graft occlusion	0	1 (8.5%)	2 (16.6%)	3 (12%)	4 (22%)	5 (50%)	N/A	N/A	0	0	11 (38%)	5 (25%)	1 (11%)	54 (53.5%)	3 (6%)
Amputation	0	N/A	0	N/A	0	N/A	N/A	1 (12.5%)	0	0	5 (17%)	1 (5%)	N/A	N/A	1 (2%)
Related mortality	0	0	2 (16.6%)	5 (20%)	2 (11%)	1 (10%)	1 (9%)	1 (12.5%)	0	4 (50%)	6 (20.5%)	1 (5%)	3 (33%)	14 (14%)	4 (8%)
Mean follow-up (months)	28.9	17	45.5	24	54.4	133.9	20	14	18	23	83.9	44	36	13	N/A

*N/A* Not available

## Conclusions

We may conclude that prosthetic wrapping for vascular suture line anastomoses in patients with Behçet’s aortic/aortoiliac aneurysms is a simple, efficient, reliable, and safe technique. It may be useful to obtain good hemostasis on the anastomotic suture line, with low morbidity and mortality rates. Moreover, this procedure was performed as a prophylactic measure to avoid the development of the postoperative pseudoaneurysms. Furthermore, the administration of adjunctive pre- and postoperative systemic immunosuppressive agents should be considered as an important factor for prophylaxis and prevention of vascular suture line anastomotic pseudoaneurysms and other graft-related complications with a long-term patency rate. It can be assumed that surgical intervention should be took place in addition to perioperative immunosuppressants.

## Abbreviations

AAA: Abdominal Aortic Aneurysm (AAA)
CBAS: Carmeda BioActive Surface
CDUS: Color Doppler Ultrasonography
CTA: Computed Tomography Angiography
DVT: Deep Vein Thrombosis
ePET: Expanded Polyethylene Terephthalate
ePTFE: Expanded Polytetrafluoroethylene
ESR: Erythrocyte Sedimentation Rate
EULAR: European League Against Rheumatism
HLA: Human Leucocyte Antigen
ICU: Intensive Care Unit
IRB: Institutes’ Review Board
PAD: Peripheral Arterial Disease
SD: Standard Deviation
SPSS: Statistical Package for the Social Science
