# Correction to: Vascular suture line wrapping for aortoiliac anastomoses following open surgical repair of infrarenal Behçet’s aortoiliac aneurysms

**DOI:** 10.1186/s13023-019-1081-x

**Published:** 2019-05-13

**Authors:** Ahmed Mousa, Ibrahim Hanbal, Alaa Sharabi, Mohammed A. Nasr, Abdelfattah K. Nassar, Mai A. Elkalla

**Affiliations:** 1 Department of Vascular & Endovascular Surgery, Al-Hussain University Hospital, Faculty of Medicine for Males, Al-Azhar University, Darrasa, Cairo, 11675 Egypt; 2 Division of Vascular & Endovascular Surgery, Department of Surgery, College of Medicine, King Faisal University, Al-Ahsa, Eastern Province 31982 Saudi Arabia; 3 Division of Vascular & Endovascular Surgery, Department of Surgery, Faculty of Medicine, Al-Azhar University, Assiut Branch, Assiut, Egypt; 4 Department of Rheumatology and Rehabilitation, Al-Hussain University Hospital, Faculty of Medicine for Males, Al-Azhar University, Cairo, Egypt; 5 Medical Student, Faculty of Medicine, Helwan University, Cairo, Egypt

**Correction to: Orphanet J Rare Dis**

**https://doi.org/10.1186/s13023-019-1048-y**

Following the publication of this article [1], the authors informed us of a typographical error in the spelling of “ePET-Dacron®” in the Background section. The corrected sentence is therefore: “While anastomosing graft to host artery, vascular suture lines has been reinforced with expanded polyethylene terephthalate (ePET - Dacron®), polytetrafluoroethylene (ePTFE), omentum, an autogenous vein, or mesh to wrap the vascular anastomoses.”
